# *Burkholderia pseudomallei* triggers canonical inflammasome activation in a human primary macrophage-based infection model

**DOI:** 10.1371/journal.pntd.0008840

**Published:** 2020-11-02

**Authors:** Sabine Lichtenegger, Julia Stiehler, Sabine Saiger, Andrea Zauner, Barbara Kleinhappl, Claudia Bernecker, Peter Schlenke, Gabriel E. Wagner, Kathrin Krause, Magdalena Gastager, Ivo Steinmetz

**Affiliations:** 1 Diagnostic & Research Institute of Hygiene, Microbiology and Environmental Medicine, Medical University of Graz, Graz, Austria; 2 Department of Blood Group Serology and Transfusion Medicine, Medical University of Graz, Graz, Austria; 3 Friedrich Loeffler Institute of Medical Microbiology, University Medicine Greifswald, Greifswald, Germany, Current address: Max Planck Unit for the Science of Pathogens, Berlin, Germany; University of Glasgow, UNITED KINGDOM

## Abstract

Most of the current knowledge on *Burkholderia pseudomallei*-induced inflammasome activation and cell death in macrophages is derived from murine systems. Little is known about the involved bacterial structures and mechanisms in primary human macrophages. This is of particular relevance since murine and human macrophages as well as primary cells and cell lines differ in many aspects of inflammasome activation, including the proteins involved in the recognition of bacterial patterns. In this study, we therefore aimed (i) to establish an *in vitro B*. *pseudomallei* infection model with human monocyte-derived primary macrophages from single donors as these cells more closely resemble macrophages in the human host and (ii) to analyze *B*. *pseudomallei*-triggered cell death and bacterial elimination in those cells. Our results show that *B*. *pseudomallei-*infected primary human macrophages not only release the inflammasome-independent pro-inflammatory cytokines IL-8 and TNF-α, but are also engaged in canonical inflammasome activation as evidenced by caspase-1 and gasdermin D processing. Absence of the *B*. *pseudomallei* T3SS-3 needle protein BsaL, a potent activator of the canonical inflammasome, abolished lytic cell death, reduced IL-1β release, and caspase-1 and gasdermin D processing. IFN-γ, known to promote non-canonical inflammasome activation, did not influence pyroptosis induction or IL-1β release from infected primary human macrophages. Nevertheless, it reduced intracellular *B*. *pseudomallei* loads, an effect which was partially antagonist by the inhibition of NADPH oxidase. Overall, our data implicate T3SS-3 dependent inflammasome activation and IFN-γ induced immune mechanisms as critical defense mechanisms of human macrophages against *B*. *pseudomallei*. In addition, our infection model provides a versatile tool to study human host-pathogen interactions and has the potential to elucidate the role of human individual genetic variations in *B*. *pseudomallei* infections.

## Author summary

Considering the constantly emerging antibiotic resistance of pathogens, comprehensive analyses of immune response mechanisms against infections are urgently needed to provide the basis for novel therapeutic strategies. Studies based on primary murine cells and cell lines of murine and human origin led to advances in the understanding of immune defense mechanisms against bacterial infections including *B*. *pseudomallei*. Nevertheless, results relying on these cell types are not always transferrable to primary human cells due to e.g. pathway alterations. We established and validated a macrophage-based model system derived from human peripheral blood monocytes, which yields high amounts of genetically identical cells more closely resembling cells found in the human host. This model system provides the basis for studying the pathogenesis of *B*. *pseudomallei* in primary human macrophages and for developing new human host directed therapies avoiding pitfalls from cell lines. Using our newly established model we demonstrate, that restriction of *B*. *pseudomallei* by primary human macrophages is mediated by T3SS-3 dependent canonical inflammasome activation and IFN-γ induced intracellular bacterial restriction.

## Introduction

Globally, a huge population is constantly exposed to the melioidosis causing environmental pathogen *Burkholderia pseudomallei* [1–5]. This results in a high proportion of asymptomatic infections as evidenced by serological studies or a broad clinical spectrum involving virtually any site [3, 6]. Severe melioidosis with bacteremia is associated with a high case fatality rate still reaching 40%, even if currently available antibiotic treatment is provided [2]. In addition to the burden melioidosis poses on endemic countries, *B*. *pseudomallei* emerged as an excellent model system to study innate immune mechanisms and host-pathogen interactions for intracellular Gram-negative infections. Comprehensive analyses of the human immune responses involved in those infections are urgently needed to provide the basis for novel therapeutic strategies.

Macrophages are at the front line of immune defenses against microbial pathogens and were shown to be critical for the protection from melioidosis [7]. Most of our knowledge about the interaction of *B*. *pseudomallei* with macrophages results from studies using cell lines or primary murine macrophages, both being highly informative for a wide range of research questions. Nevertheless, both cell sources have their limitations and some findings based on these models might not be transferable to primary human cells and human infections. Additionally, these models do not provide a tool to investigate genetic diversity in humans, which is suggested to affect innate immunity [8].

With regard to intracellular pathogen recognition there are striking differences between murine and human cells. *B*. *pseudomallei* invades the cytosol of macrophages, where it becomes exposed to inflammasomes [9–11]. These protein platforms recognize conserved bacterial structures via NOD-like receptors (NLR). One NLR, which is attributed with a role in murine melioidosis is NLRC4, which detects components of type-3-secretion systems (T3SS) of Gram-negative bacteria [12, 13]. NLRC4 depends on NAIP, a helper NLR [14–16]. Mice encode different forms of NAIP, each recognizing a different bacterial structure. NAIP1 detects the T3SS needle, NAIP2 the T3SS rod, and NAIP5 and NAIP6 flagellins [14, 16, 17]. In contrast, humans only encode one single NAIP, which is suggested to recognize components of bacterial T3SS as well as flagellins [18].

Upon recognition of bacterial structures, canonical inflammasomes activate the cysteine protease caspase-1, resulting in the induction of the highly inflammatory cell death pyroptosis and the processing of pro-IL-1β and pro-IL-18 in their active forms [12, 19, 20]. Cell lysis as a consequence of pyroptosis eliminates the intracellular replication niche of the bacteria thereby exposing them to e.g. neutrophils, and furthermore results in a massive release of IL-1β and IL-18 in the extracellular space. Both pro-inflammatory cytokines are increased in septic melioidosis, but appear to have controversial effects [13, 21–23]. While IL-18 is suggested to have a protective role by inducing IFN-γ secretion, IL-1β is associated with a detrimental effect [13, 21].

In the cytosol of murine macrophages, *B*. *pseudomallei* additionally becomes exposed to caspase-11, which is suggested to directly recognize lipopolysaccharide (LPS) without the need for a NLR. Caspase-11 was shown to be important in the protection from murine melioidosis [24, 25]. Nevertheless, this immune pathway genetically differs between mice and humans. Humans encode two orthologues of caspase-11, caspase-4 and caspase-5 [26–28]. The expression of murine caspase-11 and human caspase-5 are induced by IFN-γ [29, 30], whereas caspase-4 expression and activity appears to be independent of IFN-γ priming [31].

Inflammasome activation and responses not only differ between mice and humans, but also significantly differ between human cell lines and primary cells. Studies on the NLRC4/NAIP inflammasome using monocyte tumor cell lines suggest that in contrast to murine NAIP, human NAIP (hNaip) cannot detect cytosolic flagellin of various bacteria [16, 17, 32]. These studies used THP-1 and U937, which show reduced expression of hNAIP compared to primary human macrophages, which in turn renders them insensitive to flagellin [33]. A more recent study by Kortmann et al. revealed that *Salmonella* induces cell death and IL-1β release in primary human macrophages in a flagellin dependent manner, highlighting the fact that studies using cell lines need to be reevaluated in primary human cells [33].

Surprisingly, there are not only discrepancies between murine and human cells as well as cell lines and primary cells, but also between primary human monocytes and primary human macrophages. While LPS of Gram-negative bacteria acts as sole stimulus for caspase-1 activation and IL-1β release via the NLRC3 inflammasome in primary human monocytes, it is unable to promote these processes in fully differentiated macrophages without a second stimulus [34, 35].

Unquestionable, profound knowledge was gained from studies using primary murine cells as well as murine and human cell lines. Nevertheless, the above described differences emphasize the need for standardized primary human cell models and reevaluation of results gained using murine or cell line-based systems. The validation of immune mechanisms in primary human cells will also aid in the verification and identification of human genetic variations resulting in different susceptibilities to infections. In the present study, we (i) aimed to establish a standardized experimental system for the isolation and storage of high numbers of primary human monocytes, their differentiation to monocyte-derived primary human macrophages (hMDMs) and (ii) to investigate *B*. *pseudomallei* induced cell death and bacterial restriction.

Using our model system, we show that hMDMs release IL-1β and engage in pyroptosis in response to *B*. *pseudomallei* infections. Lytic cell death and IL-1β release are associated with canonical inflammasome activation as (i) caspase-1 processing correlated with these two phenotypes and as (ii) deletion of the canonical inflammasome inducer BsaL [18] completely abolished LDH release and massively decreased IL-1β levels in the supernatant. Further, we elucidate an important role of IFN-γ mediated immune mechanisms in the restriction of the pathogen.

## Materials methods

### Ethics statement

Blood for the isolation of primary human monocytes was obtained from healthy donors during a platelet apheresis donation. Only pseudonymized residual material from donors, who signed a regular written informed consent form for blood donation, was used. The written informed consent covers the performed basic research applications and is in accordance with the Ethical Review Committee of the Medical University of Graz.

### Bacterial strains and culture conditions

All experiments using *B*. *pseudomallei* were performed with the wild-type strain E8, a soil isolate from Northeast Thailand or an isogenic Δ*bsaL* mutant. Bacteria were grown aerobically on Columbia agar containing 5% sheep blood (BD Biosciences, Austria) at 37°C for 16 hours prior to infection experiments. For colony forming units (CFU) determination bacteria were plated on Luria-Bertani (LB) agar (Carl Roth GmbH + Co. KG, Austria) and incubated at 37°C with aeration for 48 hours.

### Targeted mutagenesis of *bsaL*

A markerless *B*. *pseudomallei bsaL* mutant strain was generated as previously described [12, 36]. Briefly, a BPSS1548 knockout construct was cloned into the vector pEXKm5 (for oligonucleotides see Table 1). Subsequently, the vector was introduced into *E*. *coli* RHO3 via heat shock transformation followed by conjugation with *B*. *pseudomallei* E8 to allow allelic replacement by homologous recombination. A sucrose counter-selection step was performed to eliminate the pEXKm5 plasmid backbone and resulting colonies were screened to identify *bsaL* mutants.

**Table 1:** Oligonucleotides used in this study.

Oligo name	Sequence (5´- 3´)
BPSS1548up_for	CTC AGG CGT CAG CGC GAG
BPSS1548up_rev BamHI	AGC TAG CTA GGA TCC CGG TAA TGT GCT CCT TCC AG
BPSS1548dn_for BamHI	AGC TAG CTA GGA TCC CCC TCT CTA CCG CTC CGG
BPSS1548dn_rev	CGG ATG CAT CGA AGG CGT C
BPSS1548ko-seq_for EcoRI	AGC TAG CTA GAA TTC GCG TCA GCG CGA GCC ATG
BPSS1548ko-seq_rev EcoRI	AGC TAG CTA GAA TTC CGT GCA TCC GCG CGT CTG

### Bacterial growth analyses

To control for any direct anti-bacterial effects of substances used in our infection experiments (see below) bacteria were grown on Columbia agar containing 5% sheep blood (BD, Austria) at 37°C for 16 hours and adjusted to an OD_600_ of 0.05 in RPMI-1640 medium in a multiwell culture plate (48-wells). Although RPMI 1640 medium is not specifically designed for bacterial growth, it was chosen, as this medium was used in our infection assays. Bacteria were incubated with recombinant human IFN-γ (5 ng/ml, 50 ng/ml or 500 ng/ml, Peprotech, Germany), aminoguanidine (2 mM, Sigma Aldrich, Austria), apocynin (500 μM, Sigma Aldrich, Austria), superoxide dismutase (SOD, 20 U/ml, Sigma Aldrich, Austria), catalase (200 U/ml, Sigma Aldrich, Austria), and *N*-acetyl-cysteine (NAC, 200 μM, Sigma Aldrich, Austria) or the corresponding vehicle at 37°C with shaking at 220 rpm in a microplate reader (Spark Multimode Microplate Reader, Tecan). The OD_600_ of bacterial cultures was measured every hour.

### Isolation of primary human monocytes

Human peripheral blood monocytes from healthy donors were obtained from the leukocyte reduction chamber of a platelet apheresis donation. Voluntary donors were accepted in accordance with the Austrian Blood Donation Ordinance. All male and female donors were within an age range of 18 and 60 years and weighed more than 55 kg, but were not obese. Monocytes were isolated by negative selection using the RosetteSep Human Monocyte Enrichment Cocktail (Stemcell Technologies, Germany) according to the manufacturer´s instructions. The purity of CD14-positive monocytes was >80% as assessed by flow cytometry. About 10^8^ cells from a single donor were cryopreserved in the corresponding medium supplemented with 10% DMSO.

### Macrophage differentiation

**Fig 1:**
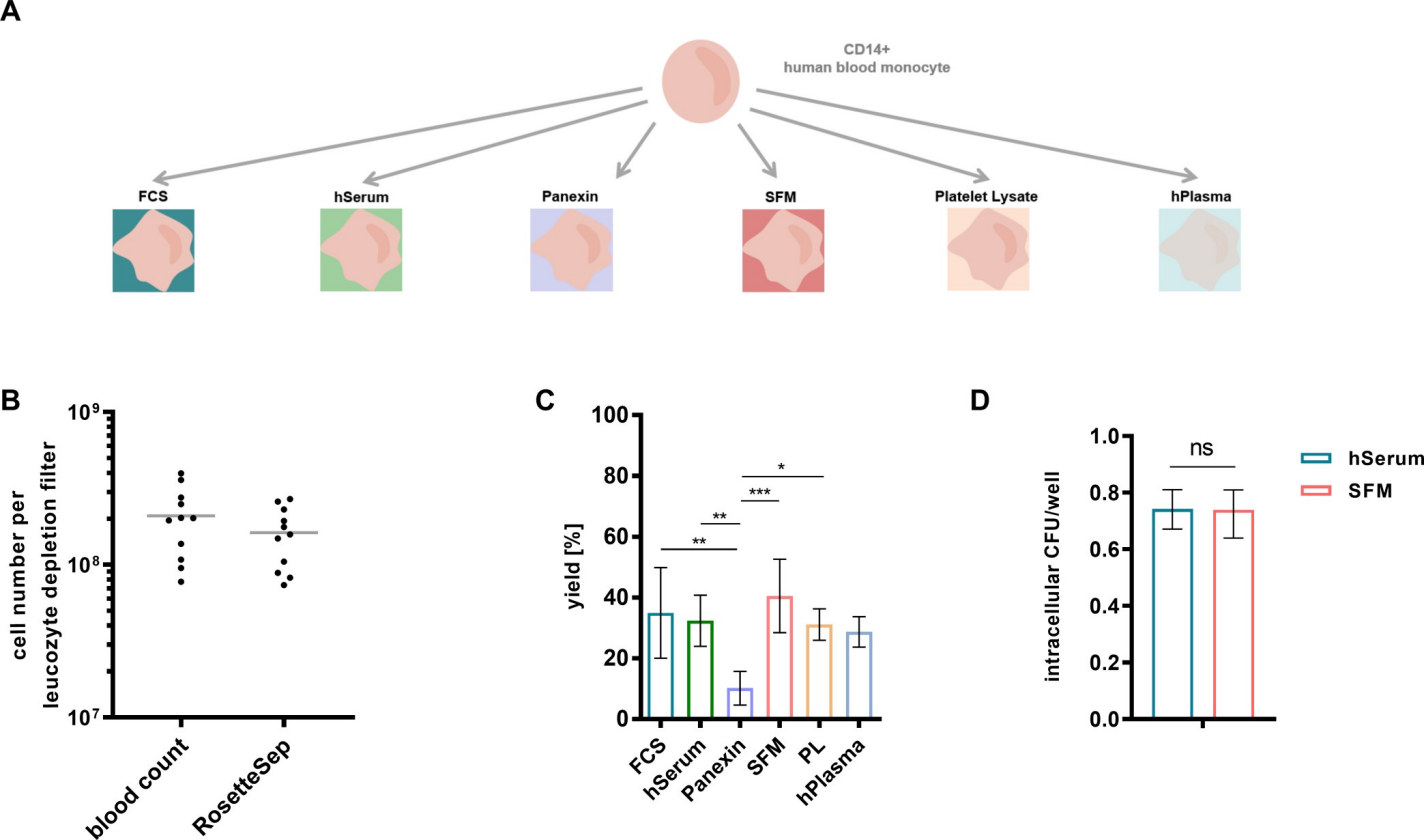
Different hMDM cultivation strategies do not affect intracellular survival. (A) After enrichment, monocytes were cultivated in FCS, hSerum, Panexin, SFM, PL or hPlasma as illustrated. (B) Human monocytes were obtained from leukocyte depletion chambers by negative selection using the RosetteSep kit. Shown are absolute monocyte numbers before (blood count) and after purification (RosetteSep) of 11 independent measurements (mean). (C) Human monocytes were differentiated into hMDMs using different serum components. Yield in % from initially seeded cells. Data are depicted as median and interquartile range (n≥4 independent experiments with different donors). Significant differences between data sets are marked by asterisks (*p < 0.05, **p < 0.01, ***p < 0.001). (D) hMDMs were infected with *B*. *pseudomallei* at an MOI of 300. Shown is the ratio of intracellular CFU/well at 3h compared to time point 0h of 3 independent experiments with different donors, performed in technical triplicates (median and interquartile range). FCS (Fetal Calf Serum), hSerum (human Serum), SFM (Serum Free Medium), PL (Platelet Lysate), hPlasma (human Plasma), hours (h), ns (not significant).

After enrichment of CD14-positive cells, primary human monocytes were either cultivated in serum-free medium (SFM, PAN-Biotech, Germany) or in RPMI-1640, both supplemented with 100 μg/ml granulocyte-macrophage colony-stimulating factor (GM-CSF, Peprotech, Germany). RMPI-1640 was additionally supplemented with one of the following ingredients: 10% heat-inactivated fetal calf serum (FCS, Life Technologies, Austria), human heat inactivated AB serum (hSerum, Sigma Aldrich, Austria), Panexin basic (PAN-Biotech, Germany), platelet lysate (PL, BioScience GmbH, Germany), or human AB plasma (hPlasma, Octapharma, Austria) (scheme shown in **Fig 1A**). Cells were cultured for 6 days at 37°C in a humidified atmosphere containing 95% air and 5% CO_2_ with medium replacement at day 3. The yield of hMDMs was determined by cell count of CD11b positive cells on day 6.

### Macrophage infection

One day prior to infection with *B*. *pseudomallei*, hMDMs were seeded in 48-well-plates at 1.3 × 10^5^ cells per well. Where applicable hMDMs were treated with IFN-γ (5 ng/ml, 50 ng/ml or 500 ng/ml recombinant human IFN-γ, Peprotech, Germany) 24 hours prior to infection. For inhibition of nitric oxide synthase (iNOS) or NADPH oxidase hMDMs were treated with 2 mM aminoguanidine (Sigma Aldrich, Austria) or 500 μM apocynin (Sigma Aldrich, Austria) for one hour prior to infection. All media used during the infection experiment were supplemented with the mentioned inhibitors and the corresponding vehicles throughout the experiment. For infection with *B*. *pseudomallei*, bacteria were adjusted to the desired concentration and added to the cells at the indicated multiplicity of infection (MOI). Cells were infected for 30 minutes (min) before hMDMs were washed and incubated with kanamycin (250 μg/ml) containing medium. At defined time points (time point 0 corresponds to 30 min after the addition of kanamycin) hMDMs were lysed with 20% saponin (Sigma Aldrich, Austria) and CFU per well were determined by plating serial dilutions on LB agar.

### Cytotoxicity assay

Cells were infected as described above and loss of cellular membrane integrity was measured by lactate dehydrogenase (LDH) release in the supernatant. LDH release was quantified using the CytoTox-One homogenous membrane integrity assay (Promega, Austria) as previously described [37]. A ratio was calculated by normalizing infected samples to their corresponding not infected control (set to 1).

### Flow cytometry

The purity of monocytes or hMDMs was examined by determination of CD14 or CD11b positive cells, respectively. Nonspecific antibody binding was blocked by the addition of FcR Blocking Reagent (BD Biosciences, Germany). Fc receptors were blocked according to the manufacturer´s protocol and cells were stained with 5 μl CD14-APC (Clone M5E2) or CD11b-PE (Clone D12) (BD Biosciences, Germany) or the appropriate isotype control. Marker expression was examined on a BD FACSCalibur flow cytometer (BD Biosciences, Germany) and data were analyzed using the BD CellQuest Pro software (Version 6.0). Living macrophages were gated according to their forward- and side scatter characteristics.

### Measurement of cytokines

Cells were infected as described above and cytokine concentrations were determined in cell culture supernatant using the Bio-Plex Pro Human Cytokine Group I Panel, 17-plex, the Bio-Plex Pro human Cytokine Group I, IL-1β or the Bio-Plex Pro human Cytokine Group I, TNF-α singleplex (BioRad, Austria) according to the manufacturer`s protocols. Data were generated on a Bio-Plex 200 system (BioRad, Austria), and data were analyzed using the Bio-Plex Manager Software (Version 6.1) (BioRad, Austria).

### Protein extraction and western blot analysis

24 hours prior to infection, hMDMs were seeded in 6 well-plates (6.5×10^5^ cells per well), and infected for 30 min with *B*. *pseudomallei* wild type or the corresponding Δ*bsaL* mutant at the indicated MOI. Proteins of hMDMs were prepared by TRIzol Reagent (ThermoFisher, Austria) according to the manufacturer's instructions. Protein content was determined using the Bradford method. Equal amounts of protein were separated by SDS-PAGE and transferred to PVDF membranes. As a loading control, all blots were probed with anti–β-actin (8H10D10, Cell Signaling). Primary antibodies against human caspase-1 (PA517570, Invitrogen), IL-1β (8516, R&D Systems), β-actin (8H10D10, Cell Signaling) and gasdermin D (NBP2-33422, Novus Biologicals) and anti-rabbit IgG (Cell Signaling) or anti-mouse IgG (Cell Signaling) HRP-conjugated secondary antibody were used. For detection, Clarity Western ECL Substrate (BioRad, Austria) was used as the HRP substrate.

### Statistical analyses

If indicated, results were tested for statistical significance using RM one-way ANOVA, one-way ANOVA (if data were already normalized) or two-way ANOVA (if response is affected by two factors) and Bonferroni’s test to correct for multiple comparisons if multiple genotypes, stimuli, or conditions were to be compared. If two genotypes, stimuli or conditions were to be compared paired t test was used. Graphing of data and statistical analyses were performed using the GraphPad Prism software (version 8.3.1). For experiments with primary human cells, at least three different donors were used. Differences were considered statistically significant if a *P* value of <0.05 was determined.

## Results

### Different hMDM cultivation strategies do not affect *B*. *pseudomallei* intracellular survival

The isolation of CD14 positive monocytes from leukocyte depletion chambers by the used RosetteSep kit resulted in high monocyte yields of about 10^8^ cells from a single donor (Fig 1B).

After maturation, the hMDMs yield was about 4*10^6^ from initially 1*10^7^ seeded cells for serum free medium and RPMI-1640 supplemented with FCS, hSerum, platelet lysate or hPlasma (Fig 1C). Only panexin basic, a serum replacement with defined components, yielded lower amounts of macrophages (2*10^6^) (Fig 1C). Cell viability was not affected by the different cultivation strategies as analyzed by PI staining and flow cytometry.

As human serum contains e.g. cytokines, which could influence the bactericidal activity of cells, we infected hMDMs cultivated with human serum and hMDMs cultivated in SFM with *B*. *pseudomallei*. Fig 1D shows that the bactericidal activity of hMDMs against *B*. *pseudomallei* was similar for both conditions.

Overall, hMDMs differentiated in hSerum yield high numbers of viable macrophages, which do not show increased bactericidal activity compared to macrophages differentiated in a chemically defined component. Therefore, we decided to use hMDMs differentiated in hSerum for all subsequent experiments.

### *B*. *pseudomallei* leads to inflammasome activation in hMDMs

**Fig 2:**
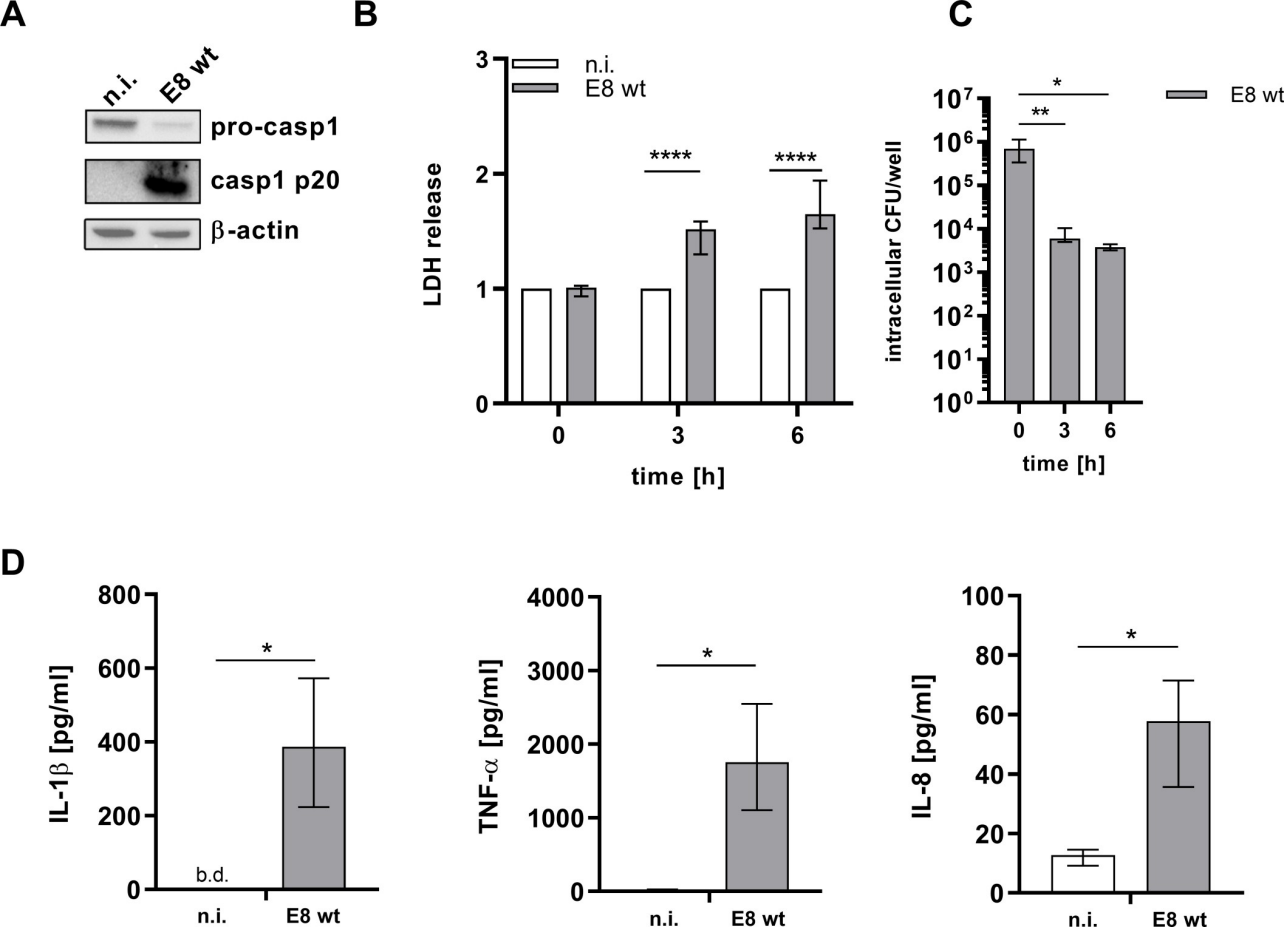
*B*. *pseudomallei* leads to inflammasome activation in hMDMs. hMDMs were infected with *B*. *pseudomallei* E8 (MOI 300) for 3 or 6h. (A) Immunoblot analysis was performed on lysates (3h p.i.) for full-length caspase-1 (pro-casp1) and cleaved caspase-1 (casp1 p20). Lysates were re-probed for β-actin. One representative experiment of three with different donors is shown. (B and C) Cell death and intracellular bacterial burden (in CFU/well) were determined at 0, 3 and 6h p.i. Shown are the results of at least 5 independent experiments with different donors performed in technical duplicates (median with interquartile range). (D) Cytokine release in supernatants was measured 3h p.i. Shown are the results of 4 independent experiments with different donors performed in technical duplicates. Data are represented as median with interquartile range. (*p < 0.05, **p < 0.01, ****p < 0.0001). caspase-1 (pro-casp1), cleaved caspase-1 (casp1 p20), n.i. (not infected), wt (wild type), b.d. (below detection), hours (h), p.i. (post infection).

Considering the important role of inflammasome dependent immune mechanisms as response to Gram negative bacterial infections, we first utilized our established model to investigate whether *B*. *pseudomallei* infection results in the activation of caspase-1, the effector molecule of canonical inflammasomes. As evident from Fig 2A *B*. *pseudomallei* triggers caspase-1 cleavage at 3h post infection. Caspase-1 activation correlates with the induction of pyroptosis as measured by LDH release (Fig 2B). This coincides with a decrease in intracellular CFU at the indicated time points (Fig 2C). Additionally, macrophages respond to a *B*. *pseudomallei* infection with the release of IL-1β and the pro-inflammatory cytokines TNF-α and IL-8 (Fig 2D).

**Fig 3:**
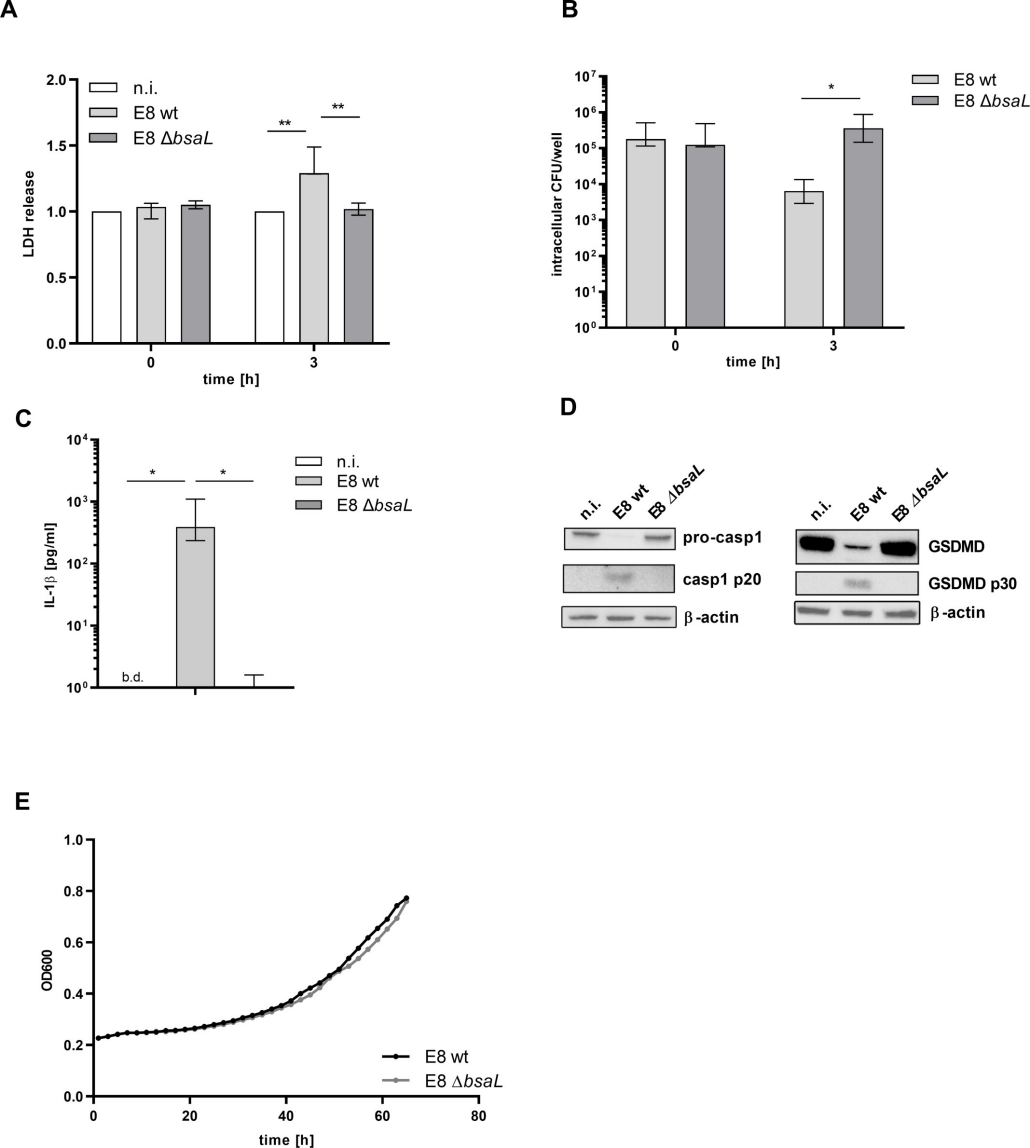
Pyroptosis and IL-1β release is dependent on the *B*. *pseudomallei* T3SS-3. hMDMs were infected with *B*. *pseudomallei* (MOI 300) for 3h. (A and B) Cell death induction and intracellular bacterial burden were determined at 0 and 3h p.i. Shown are the median and interquartile range of at least three independent experiments with different donors performed in technical duplicates. (C) IL-1β secretion was determined 3h p.i. Data are presented as median and interquartile range of four independent experiments with different donors performed in duplicates. (D) Caspase-1 and gasdermin-D processing were investigated 3h p.i. Lysates were re-probed for β-actin. For immunoblot analysis one representative experiment of at least three with different donors is shown. (E) Growth analysis of wt and Δ*bsaL* was performed. Shown are mean values of three independent experiments performed in technical triplicates. (*p < 0.05, **p < 0.01). n.i. (not infected), wt (wild type), b.d. (below detection), hours (h), p.i. (post infection), GSDMD (gasdermin D).

Fig 3A shows that infection of hMDMs with a *bsaL* deletion mutant leads to LDH levels comparable to the not infected control and to increased *B*. *pseudomallei* loads at 3h p.i. (Fig 3B). In addition to a decrease in LDH release, IL-1β secretion is drastically reduced in Δ*bsaL* infected hMDMs compared to wild type infected immune cells (Fig 3C). Correlating with the reduced LDH release and IL-1β secretion, caspase-1 cleavage in *bsaL* mutant infected cells decreases to a not detectable level when compared to the wild type (Fig 3D, see S1 Fig for all donors analyzed). We demonstrate that gasdermin D is cleaved in wild type infected hMDMs, while there is no cleavage product detected in those infected with the mutant (Fig 3D, see S1 Fig for all donors analyzed). To investigate whether these observations result from an altered growth phenotype of the mutant strain compared to the isogenic wild type, we performed growth analysis. As evident from Fig 3E the *bsaL* mutant does not show growth alterations compared to the wild type.

Overall, we could demonstrate that *B*. *pseudomallei* induces T3SS-3 dependent inflammasome activation in primary human macrophages, resulting in pyroptosis and IL-1β release.

### IFN-γ contributes to cytosolic restriction of *B*. *pseudomallei* in hMDMs

**Fig 4:**
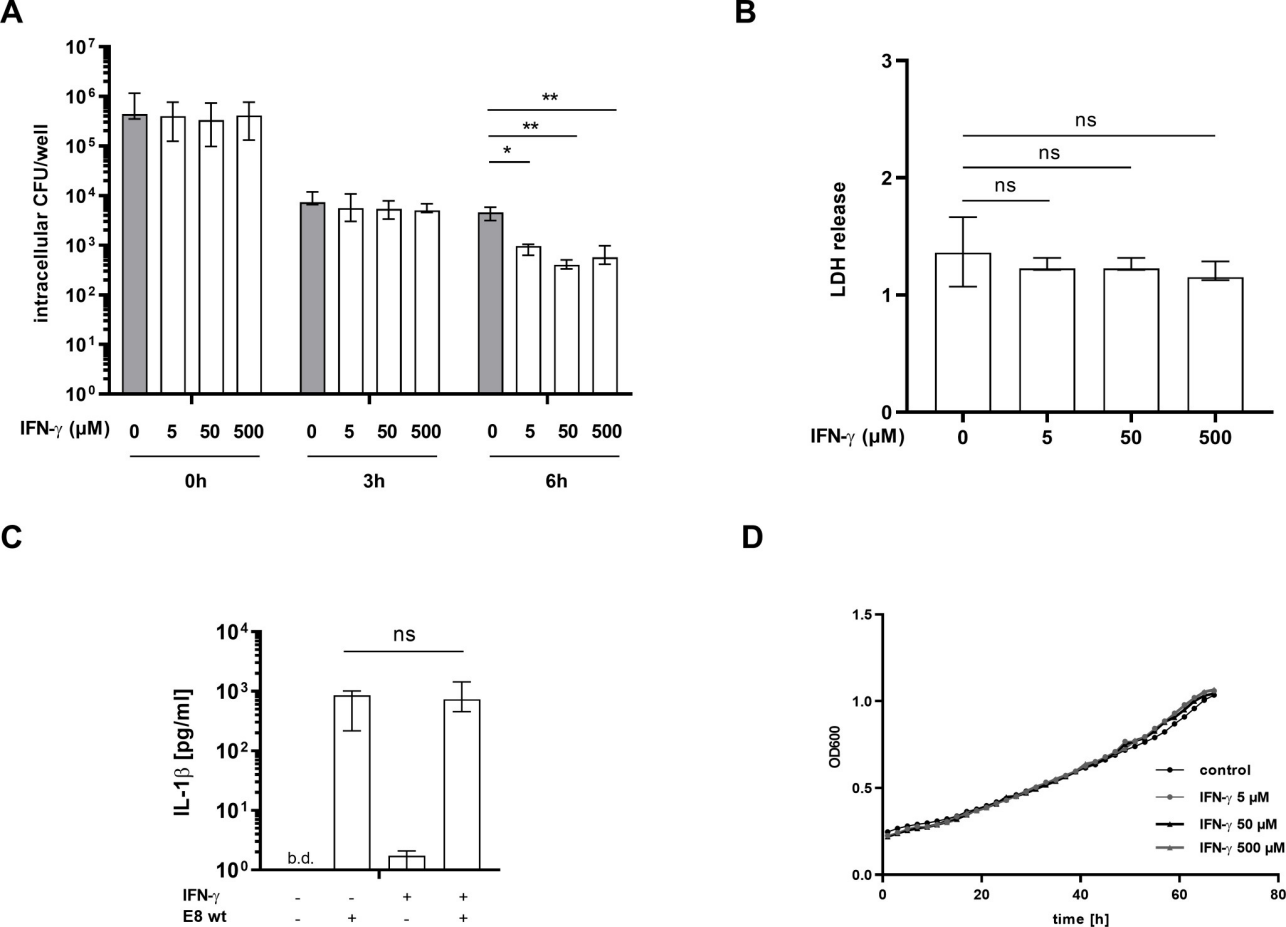
IFN-γ contributes to cytosolic restriction of *B*. *pseudomallei* in hMDMs. hMDMs were infected *with B*. *pseudomallei* (MOI 300) in the absence or presence of IFN-γ (5, 50 or 500 μM). (A) Cells were lysed 0, 3 or 6h p.i. and intracellular bacterial burden was measured. Graphs show the median with interquartile range of four independent experiments with different donors performed in duplicates. (*p < 0.05, **p < 0.01). (B) Induction of pyroptosis was measured by LDH release in supernatants 6h p.i. Graphs show the median with interquartile range of three independent experiments with different donors performed in duplicates. (C) IL-1β release was measured 6h p.i. in the presence or absence of 500 μM IFN-γ. Graphs show the median with interquartile range of three independent experiments with different donors performed in duplicates. (D) Growth analysis of the wt in media with different concentrations of IFN-γ was performed. Data are represented as mean of three independent experiments performed in duplicates. wt (wild type), b.d. (below detection), hours (h), p.i. (post infection), ns (not significant).

Next, we wanted to address the effect of IFN-γ on hMDMs, as IFN-γ is an essential innate immune component in the response to a *B*. *pseudomallei* infection. Therefore, we analyzed the bacterial burden in IFN-γ stimulated and unstimulated hMDMs. Initial uptake of *B*. *pseudomallei* and bacterial burden after 3 hours was comparable in stimulated and unstimulated cells (Fig 4A). After 6 hours of infection, IFN-γ stimulated hMDMs showed significantly lower intracellular counts of bacteria compared to the vehicle treated control. Performing growth analysis, we could exclude a direct effect of IFN-γ on bacterial growth (Fig 4D). These results indicate that IFN-γ mediated immune mechanisms are crucial for the defense of hMDMs against *B*. *pseudomallei* and suggest a role of IFN-γ in the protection from human melioidosis. To investigate whether the decreased bacterial burden of IFN-γ pretreated hMDMs results from pyroptosis induction, we measured LDH release of infected cells and its dependence on IFN-γ stimulation. As evident from Fig 4B and Fig 4C, exogenous IFN-γ treatment did neither result in altered pyroptosis induction nor IL-1β release. Overall, our data indicate that the observed IFN-γ mediated effect on intracellular bacterial loads does not result from pyroptosis induction in hMDMs, but enhancement of their bactericidal activity.

### IFN-γ mediated bactericidal activity of hMDMs is dependent on ROS generation

**Fig 5:**
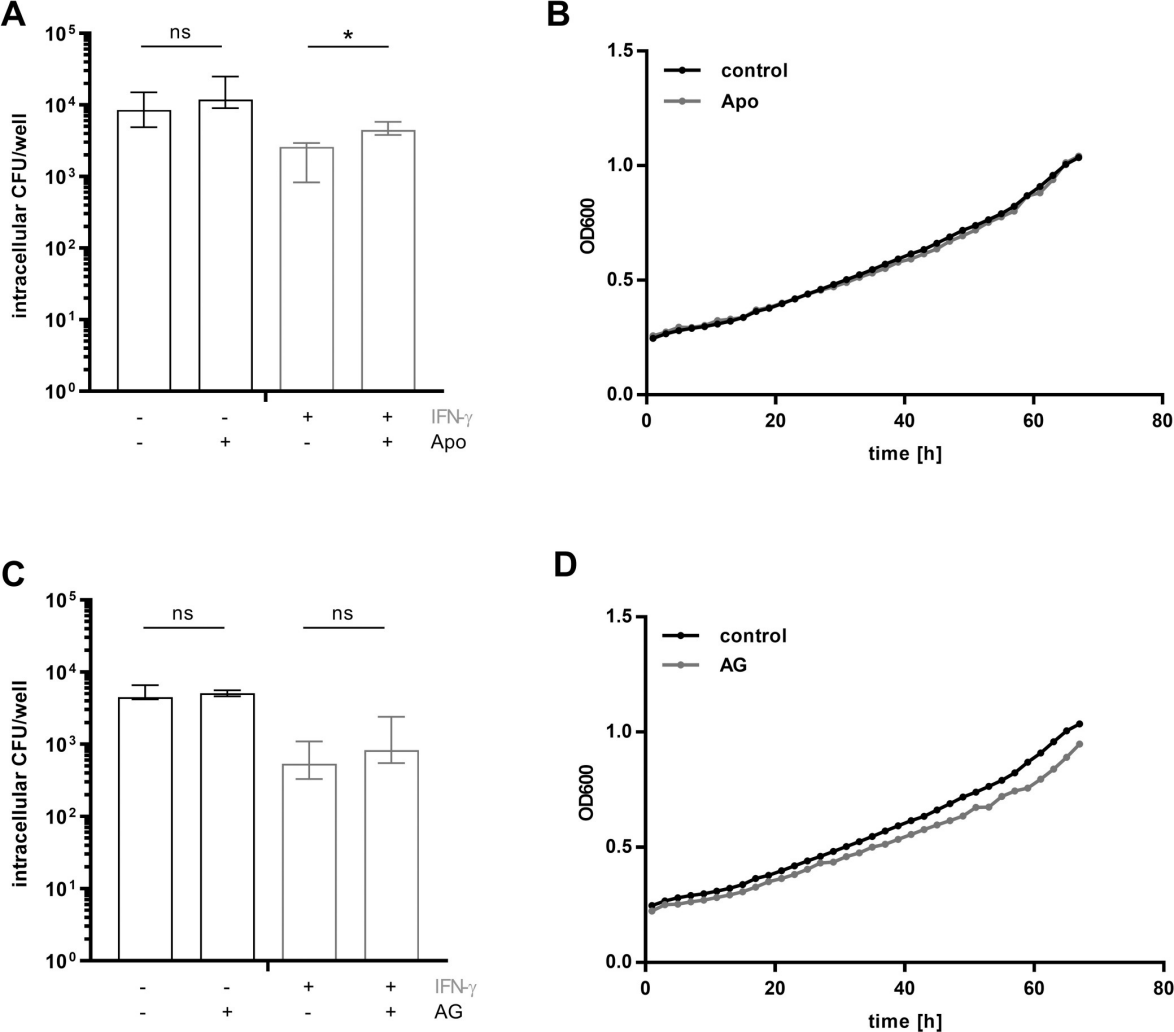
The antimicrobial effect of IFN-γ is mediated by the production of ROS. hMDMs were incubated with 500 μM IFN-γ and inhibitors of inducible NADPH oxidase (apocynin) (A) or nitric oxide synthase (aminoguanidine) (C) prior to and during the infection with *B*. *pseudomallei* (MOI 300). The intracellular bacterial burden was determined 6h p.i. Data are expressed as median with interquartile range of ≥ 3 independent experiments with different donors. All experiments were performed in technical duplicates. (*p < 0.05) To determine an effect of the corresponding inhibitors on *B*. *pseudomallei*, growth curves were performed in control medium and medium containing Apo (B) or AG (D). Shown are mean values of 3 independent experiments performed in duplicates. Apo (apocynin), AG (aminoguanidine), p.i. (post infection), h (hours), ns (not significant).

IFN-γ induces several important microbicidal mechanisms, including NADPH oxidases and iNOS. Both were previously shown to be important immune defense mechanisms against *B*. *pseudomallei* in murine melioidosis [7, 38, 39]. Therefore, we investigated the putative role of these mechanisms in the observed IFN-γ mediated decrease in bacterial loads in our hMDM model, as shown by the results in Fig 5. hMDMs were exogenously stimulated with IFN-γ and treated with the iNOS inhibitor aminoguanidine or the NADPH oxidase inhibitor apocynin. In accordance to observations in the murine system [39], inhibition of NADPH oxidase led to increased intracellular bacteria in IFN-γ treated cells (Fig 5A), while inhibition of iNOS by aminoguanidine had no effect on IFN-γ mediated decreased bacterial burden (Fig 5C). There was no direct effect of apocynin or aminoguanidine on bacterial growth (Fig 5B and 5D).

Our results verify the importance of IFN-γ mediated defense mechanisms in the restriction of intracellular *B*. *pseudomallei* in hMDMs and strengthen the hypothesis that IFN-γ contributes to define the course of an infection.

## Discussion

Several aspects of inflammasome activation have been shown to differ depending on the macrophage model used. These discrepancies partially rely on genetic differences between murine and human cells or the expression status of inflammasome components in certain cell lines. Experiments relying on primary human cells will help to identify new therapeutic targets and immune mechanisms and to revisit targets and mechanisms which could be identified in murine or cell line models. In our study, we therefore provide a simple and robust protocol for the isolation of monocytes from leukocyte depletion chambers and their subsequent differentiation to hMDMs with high yields. The use of hSerum for the differentiation of hMDMs resembles conditions related to those macrophages naturally face. The obtained high cell number from a single donor allows the use of genetically identical cells for different assays simultaneously and for technical replicates, abolishing effects based on genetic differences between donors. On the other hand, using different donors offers the chance to identify host specific differences resulting e.g. from genetic polymorphisms which could either increase susceptibility to bacterial infection or generate resistance [8].

Inflammasome dependent cell death and IL-1β and IL-18 release are important defense mechanisms against Gram-negative bacterial pathogens. Nevertheless, both also mediate septic shock and IL-1β release is associated with a poor outcome in melioidosis infections. Therefore, now constantly reported new inhibitors of inflammasome activation represent promising intervention strategies for *B*. *pseudomallei* infections. However, the role of inflammatory caspases in the course of a *B*. *pseudomallei* infection of primary human macrophages was still unclear. Using our macrophage model we show that caspase-1 is processed in *B*. *pseudomallei* infected hMDMs, an observation not previously reported. Consistently, infected hMDMs engage in pyroptosis and IL-1β release. Our results are corroborated by studies showing canonical inflammasome activation in *B*. *pseudomallei* infected murine macrophages [12, 13, 24]. A study by Weehuizen et al. described a downregulation of monocyte caspase-1 mRNA levels in melioidosis patient with sepsis [40]. This finding together with our data indicates a potential role of caspase-1 dependent events and possible individual caspase-1 expression differences in the outcome of human melioidosis.

Next, we tested our hypothesis that pyroptosis and IL-1β release are mainly dependent on caspase-1 activation in hMDMs. As the T3SS is known to be a potent activator of the canonical inflammasome, we tested a *B*. *pseudomallei* T3SS-3 structural protein mutant (Δ*bsaL*) in our infection assays. Indeed, we could show that the mutant did not induce detectable caspase-1 processing or pyroptosis. Additionally, infection with the T3SS-3 mutant drastically reduced IL-1β release from infected cells compared to the wild type. Our results are substantiated by a recent study, which indicates a similar mechanism for recombinant expressed T3SS-3 needle proteins from its close relative *Burkholderia thailandensis* [18]. These results suggest that at least at earlier time points inflammasome induction is T3SS-3 dependent in hMDMs. The role of *B*. *pseudomallei* triggered non-canonical inflammasome activation in human myeloid cells still needs to be investigated and is currently under investigation in our laboratory.

The essential role of IFN-γ in the early control of melioidosis has been indicated by murine *in vitro* and *in vivo* experiments as well as in clinical studies [41]. Blocking of IFN-γ in a melioidosis mouse model led to a drastically reduced LD50 and increased the bacterial burden in spleen and liver [42]. Jenjaroen et al. demonstrated that T-cell derived IFN-γ is elevated in melioidosis survivors compared to fatal cases. This study also highlights the fact that the overall IFN-γ response in response to a *B*. *pseudomallei* infection is lower in patients with the main melioidosis risk factor type 2 diabetes (T2D) compared to non-T2D patients [43]. This observation is substantiated by *in vitro* studies, which show that stimulation of whole blood or PBMCs from diabetes patients induces impaired IFN-γ responses associated with reduced bacterial killing [44, 45]. To define the role of IFN-γ stimulation for the bactericidal activity of hMDMs, we pre-activated these cells with external IFN-γ. The observed increase in bactericidal activity suggests a contribution of IFN-γ mediated immune mechanisms to the restriction of the pathogen. IFN-γ secretion is induced by IL-18, a pro-inflammatory cytokine, which is processed by caspase-1. IFN-γ in turn primes caspase-11 and therefore triggers non-canonical inflammasome activation in murine cells [31]. However, our data do not indicate a crucial contribution of IFN-γ in pyroptosis induction in *B*. *pseudomallei* infected hMDMs at the investigated time points. In contrast, our data suggest an IFN-γ induced ROS dependent killing mechanism at least partially contributing to intracellular bacterial restriction. Although not previously shown for differentiated hMDMs, our findings are supported by data from studies using murine macrophages [46] murine *in vivo* models [38] and human monocytes [47]. IFN- γ is a central inducer of antimicrobial activities and is known to e.g. stimulate non-canonical autophagy in *B*. *pseudomallei* infected murine macrophages [48]. The latter could also contribute to the herein observed IFN-γ mediated bacterial restriction in hMDMs and is currently under investigation in our laboratory. In summary, we provide a protocol for the efficient generation of high numbers of genetically identical hMDMs. Based on this model we were able to identify pyroptosis induction and IFN-γ induced immune mechanisms as crucial innate immune mechanisms of *B*. *pseudomallei* infected hMDMs thereby destroying its replicative niche and restricting intracellular bacterial loads.

## Supporting information

S1 Fig Caspase-1 and gasdermin-D processing for 3 individual donors. Caspase-1 (A) and gasdermin-D (B) processing were investigated for 3 different donors 3h p.i. Lysates were re-probed for β-actin.
(TIF)
