# Supplementary figures and images for: Burkholderia pseudomallei triggers canonical inflammasome activation in a human primary macrophage-based infection model
Source: PLoS Negl Trop Dis. 2020 Nov 2;14(11):e0008840. doi: 10.1371/journal.pntd.0008840 (PMC7605897; doi:10.1371/journal.pntd.0008840)

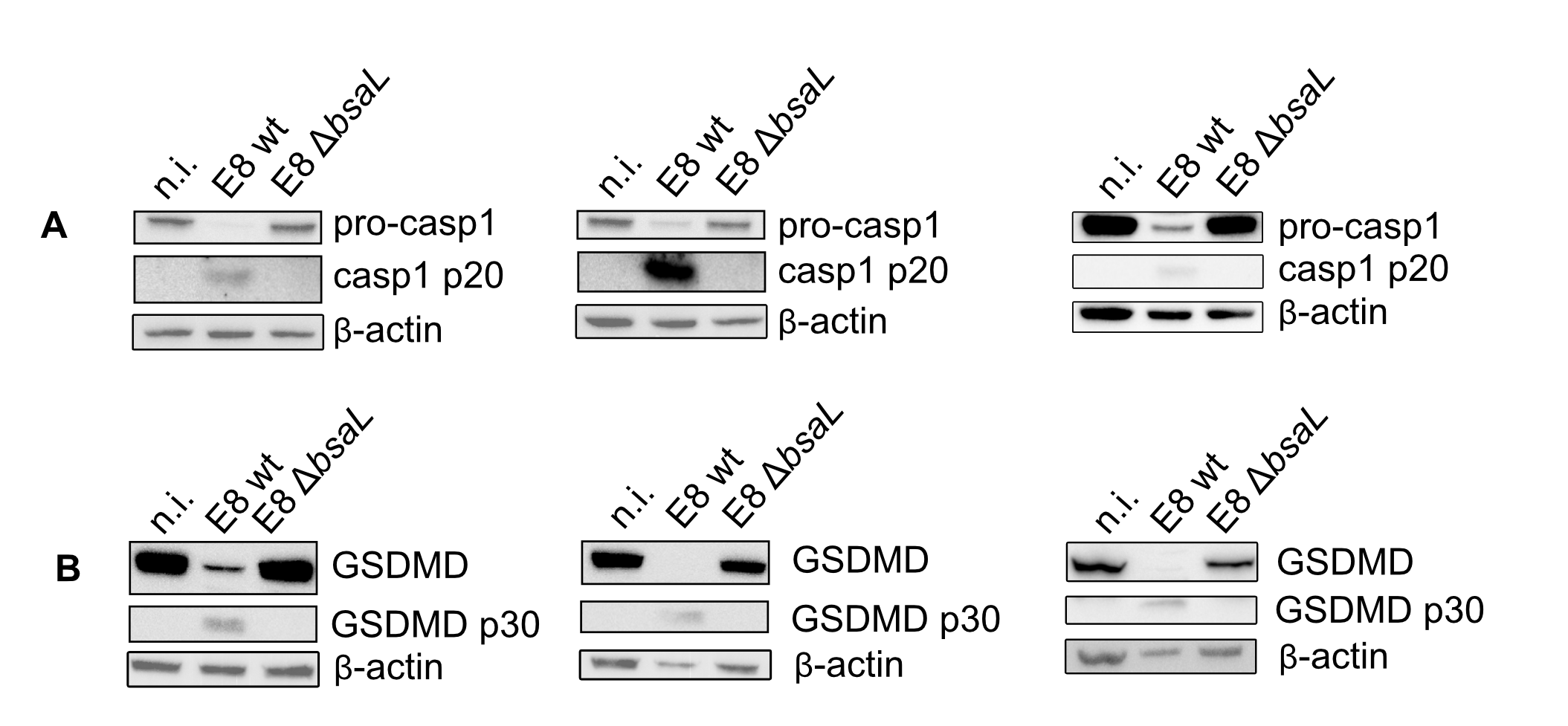

Supplement: S1 Fig — Caspase-1 (A) and gasdermin-D (B) processing were investigated for 3 different donors 3h p.i. Lysates were re-probed for β-actin. (TIF) [file pntd.0008840.s001.tif]
